# Understanding the role played by parents, culture and the school curriculum in socializing young women on sexual health issues in rural South African communities

**DOI:** 10.1080/17290376.2018.1455603

**Published:** 2018-04-05

**Authors:** Feziwe Mpondo, Robert A.C. Ruiter, Dilana Schaafsma, Bart van den Borne, Priscilla S. Reddy

**Affiliations:** a Department of Work and Social Psychology, Maastricht University, Maastricht, Netherlands *Email: feziwe.mpondo@maastrichtuniversity.nl; b Fontys University of Applied Sciences, Eindhoven, Netherlands; c CAPHRI School of Public Health and Primary Care, Maastricht University, Maastricht, Netherlands; d Population Health, Health Systems and Innovation, Human Sciences Research Council, Cape Town, South Africa; e Child and Family Studies, Social Work Department, University of the Western Cape, Cape Town, South Africa

**Keywords:** *adolescents*, *rural communities*, *communication*, *culture*, *sexual health*

## Abstract

**Background:** the decline in South Africa’s HIV infection rates especially among young women is encouraging. However, studies show that the 15–24-year-old cohort remains vulnerable. As they still report early sexual debut, being involved in sexual partnerships with older men as well as having unprotected sex. These risky sexual behaviors may be linked to factors such as the parent–child sexual health communication and the timing of the first talk. The quality of sexual health information received in school may also be important for enhancing healthier sexual behaviors. **Aims and Objectives:** to investigate the *what*, *when* and *how* sexual health communication occurs in rural South African families and to determine whether such communication patterns have changed over time. We also wanted to get an in-depth understanding of the roles played by culture, sexual health education and peers in the socialization of young women on sexual matters. **Methods:** a purposive sample of (*n = 55*) women who were 18–35 years old was selected and interviewed in focus group discussions (FGDs). **Results:** the FGD findings show that parent–child communication on sexual matters in rural communities is limited to messages that warn against pregnancy. It is also laden with cultural idioms that are not well explained. The school sexual health curriculum also fails to adequately equip adolescents to make informed decisions regarding sexual matters. All this seems to leave room for reception of misguided information from peers. **Conclusions:** findings highlight a need for designing interventions that can create awareness for parents on the current developmental needs and sexual behavior of adolescents. For adolescents programs would need to focus on providing skills on personal responsibility, and how to change behavior to enhance sexual health.

## Introduction

The decline in South Africa’s HIV infection rates especially among young women is encouraging. However, a national behavior survey still shows that 5% of 15–24-year-old females report early sexual debut that is having sex before the age of 14 years (Shisana et al., 2014). Although this rate is considerably higher in male peers (11.7%), it is still a marker for risky behavior later in life, especially in the South African context where the same survey shows a clear indication of age mixing in sexual relationships. The HIV incidence rates for 15–19-year-old females are at 5.6%, which is 2.3 times higher than male peers. A similar pattern is also observed between 20–24-year-old females (17.4%) and 24–29-year-old males (17.3%) suggesting that young women are infected by men outside their age cohort (Shisana et al., 2014). Age mixing poses a threat to young women because the same partners may be involved in sexual networks with older women, and they may also be in concurrent relationships that involve HIV transmission (Maughan-Brown, Kenyon, & Lurie, 2014; Shisana et al., 2014). In addition, a quarter of 15–24-year-old South African women report inconsistent condom use with sexual partners and 5% of these women report being involved in concurrent partnerships (Shisana et al., 2014). Furthermore, South Africa is still faced with a high prevalence of teenage pregnancy (12%), exposing these young women to more adverse health outcomes (StatsSA, 2011).

The sexual health risk factors highlighted here affect young people at a very sensitive time, when they are transitioning from adolescence into early adulthood. The developmental period involves physical and neurocognitive changes, which in turn also significantly influence psychological health and sexual behavior. Therefore, it is important to ensure that adolescents and early adults are equipped with decision-making skills that will enable them to protect themselves so that they can lead healthier lives (Sawyer et al., 2012). Studies conducted in the United States of America and in sub-Saharan Africa show that good sexual health education has a positive impact on the reproductive health of adolescents. For example, it may lead to the delaying of sexual debut and a lower number of sexual partners later (DiClemente et al., 2001; Phetla et al., 2008; Stidham-Hall, Moreau, & Trussell, 2012). However, the benefits are also strongly linked to the timing of the initial parent–child conversation, the frequency, and the content of the talks (Phetla et al., 2008; Rosenthal & Feldman, 1999).

Sexual health communication that is provided by the parents is considered very important. It is not only complimentary to the knowledge that adolescents may receive from school but it is also considered important because parents see their children daily and are better placed to reinforce important lessons (Sidze, Elungata’a, Maina, & Mutua, 2015). In addition, parents get an opportunity to pass their beliefs and values with regard to sexual matters. They are able to provide a nurturing and supportive environment while simultaneously monitoring the behavior of their children (Coetzee et al., 2014; Markham et al., 2010). While studies show that good parent–child communication is important, the same research indicates that this is not yet the norm in South Africa (Halpern-Felsher, Kropp, Boyer, Tschann, & Ellen, 2004; Phetla et al., 2008). This suggests a need to conduct research that will help us further understand the determinants of this poor communication and how these can be targeted in developing behavior change interventions (Coetzee et al., 2014; Halpern-Felsher et al., 2004; Namisi et al., 2015).

Among Black South African families, lack of communication has been attributed to the fact that parents have always viewed overt sex talk as inappropriate and therefore impermissible. This is similar to the experiences of families in other African countries. Therefore, instead of using direct speech, Black South African families would comunicate through the use of local referents. In other cases, the help of extended family members such as uncles and aunts would be enlisted (Delius & Glaser, 2002; Kajula, Darling, Kaaya, & Vries, 2016; Lambert & Wood, 2005; Nambambi & Mufune, 2011). Often, sexual health messages were relayed during traditional ceremonies known as the rite of passage. Studies also show that in India communities follow similar cultural practices (Eaton, Flisher, & Aarø, 2003; Lambert & Wood, 2005; Namisi et al., 2015; Phetla et al., 2008). The social and cultural aspects that influence this lack of communication in Black South African families, especially among those in rural areas, are not very well understood.

This study utilized a qualitative research approach to investigate *what*, *when* and *how* parent–child sexual health communication happens in rural settings. Firstly, the manner in which parents transfer sexual health information in a home setting was investigated. Secondly, the study sought to understand the use of cultural traditions in socializing young women on sexual matters. Further, this study investigated whether young women regarded the sexual health education received from school adequate for equipping them with appropriate knowledge to enact healthy behaviors. This was done through conducting focus group discussions with adolescent girls, young and adult women.

## Methods

### Study setting

This study was conducted between May 2011 and June 2011 in five villages, which collectively form the OR Tambo district in the Eastern Cape Province, South Africa. The OR Tambo district is located along the eastern seaboard of the Indian Ocean in the Eastern Cape Province. The total population of the province is 6.2 million and 78.8% of those people are Black and speak isiXhosa (StatsSA, 2014), and 11% are women between the ages of 15–35 years (DEDEAT, 2013). The province ranks second highest in food insecurity, low economic activity, wealth-inequality and unemployment. The villages sampled in the study were selected using data gathered during stakeholder meetings as well as literature reviews conducted prior to the commencement of the study. The data showed that these villages reflect the social challenges faced by the rest of the province and that households which are headed by females suffer the most from these social challenges (DEDEAT, 2013). Further, the Eastern Cape has high-prevalence rate of HIV infection (11.6%) as is true for the rest of South Africa, highlighting a need for effective behavior change interventions (Shisana et al., 2014).

### Participants

A purposive sampling method was used with the aim of getting a subset of young women who represented the profile of the rural communities outlined above. Women were recruited to participate in the study through a local tribal authority and development organization, the network of Royal Chief’s wives: Imbumba Yoomama Bakomkhulu (IYA). IYA members recruited the reseach participants for the study by using the word of mouth as well as networks within community structures. The research team then contacted women who indicated their interest and avialability to participate in focus group discussions. Participants were invited if they were 18–35 years of age, had a qualification below the South African Senior Certificate and lived in the designated area. The groups were organized according to age categories to allow ease of sharing information; we anticipated that older women would dominate conversations if age groups were mixed. This is mainly because in rural communities elders are always accorded respect by younger people. Therefore, a total of 7 focus group discussions (FGDs) with a total of 55 participants were set up. Three of the groups comprised adolescents and young adults (18–24 years; *n = 30*), and there were four groups of adult women (25–35 years; *n = 25*). Each group had 5–15 participants in total. The research team agreed that the number of FGDs held here were sufficient as research also shows that the average number for adequate data analysis is four to five (Carlsen & Glenton, 2011). All groups included a combination of those young women who had children as well as those that did not.

### Data collection and analysis

The FGDs were conducted using semi-structured interview guides (Bernard & Ryan, 2009). The FGDs were part of a needs assessment step for a planned intervention that was to be developed later. The needs assessment sought to gain an in-depth understanding of social factors that affected the health of young rural women. Therefore, experiences of women’s physical, mental, sexual and reproductive health issues were explored. The interview guides were developed based on literature review conducted earlier as well as discussions held by the research team. The research team included the principal investigator, lead researcher and two other Xhosa-speaking researchers. The outline of the questions in all the guides was the same, however, some questions were adapted to each age group.

This paper reports on the domains of sexual and reproductive health communication from the needs assessment. A Xhosa-speaking researcher trained in qualitative research conducted the FGDs, and was accompanied by two English-speaking researchers who were note takers. After each FGD a meeting took place between the research team and the principal investigator with the purpose of evaluating and improving the process. Even though the FGDs were not held in any particular order, data gathered from preceding FGDs informed subsequent ones. At the beginning of every discussion, verbal consent was sought from the participants. Ethics approval was obtained from Walter Sisulu University Ethics and Bio-safety Committee. All discussions were conducted in isiXhosa, were audio-recorded, and lasted 1 to 1 hour 45 minutes.

The audio-recorded FGDs were first transcribed into isiXhosa and then translated into English. A comparative analytic method-open, axial and selective coding based in the grounded theory was used to analyze the data (Glaser & Strauss, 1967). The lead author started by reading each transcript and writing data summaries or memos as a way of organizing noteworthy meaning units and interpreting the data. In the initial analysis phase, prominent themes began to emerge and these were categorized. The lead author first held discussions with the 2nd and 3rd author, respectively, where they sat and engaged on emerging categories, themes and sub-themes. Subsequently, meetings of similar format were held with the rest of the co-authors to check and verify the analysis process. The data coding was done using NVivo qualitative data management software (NVivo/QSR International Pty Ltd. 10.04, Doncaster, Australia).

## Results

The results reported here delineate the discussions of participants, namely the adolescents, young adults and adult women on how they experienced the transition period from adolescents to young adulthood. The emerging themes were organized as follows:

- The expected role of a mother
- Talks between adolescents, young adults and their mothers
- Adult women expect adolescents to be empowered by sexual education received in school
- How adult women received sexual health information in the past and how that influenced their behavior
- How the experiences of being a current adolescent inform sexual behavior

### The expected role of a mother

An understanding of the family set up for the teenagers and young adults had to be established at the outset (i.e. presence of father and mother). Most adolescents and young adults reported that mothers were always the active parents. For some girls fathers lived elsewhere as migrant workers, while for most participants the fathers were just absent. In the discussions adolescents and young women shared that they see their mothers as playing an important role in their lives. According to the young women (adolescents and young adults) a mother is expected to prepare her daughter for the different life stages, and equip her with life skills to deal with potentially difficult situations.

[…] She [mother] is the one who guides me, who knows me, when she tells me what I will meet in life and when I need something, she shows me that she is the parent and she teaches me how I will need to stand up for myself when I am older. (Adolescents and young adults group, Ntabankulu)

In the discussions, adolescents and young adults described the kind of ‘talks’ they received from their parents, as well as the exact period in which these occurred. They mainly explained that their mothers did not really start talking until they started menarche. Most girls and young women explained that they were the ones who reported what was happening with their bodies. Only then were they given some information on the reason for bodily changes. A minority of the adolescents reported that their mothers spoke about the stages of development prior to menarche. Some girls gave account of also learning things from talking to or overhearing their older sisters.

Yes, I told my mother that I am now at that stage [menstruating] and she told me what was happening and that I am old now and I should not sleep with boys. (Adolescents and young adults, Ntabankulu)

No one taught me but I used to hear my sisters talk and would not really pay attention, I mean I was not told at home, I just heard my sisters say something like this happens when you are older and they also never told me what to do when it happens, I stayed like that and when the time came I went to her [mother] and told her this was happening and she said that you have become older. (Adolescents and young adults, Ntabankulu)

The reports of adolescents and young women on sexual information received from their mothers indicate that when it comes to emotional issues that come with being at this stage of development are not really addressed. The adolescents expressed a strong desire for mothers to allow conversations about boys, while the young adults explained that they desire open relationships with their parents. However, most young women shared that this was not possible as parents were quick to dismiss or scold them.

It is very important for a child to listen but it is equally important for the parents to listen to their children. (Young adults group, Ngqeleni)

I think that maybe, if I just fell pregnant I would not be able to tell my mother like that because maybe she would scold me. (Young adults group, Ngqeleni)

### Talks between adolescents, young adults and their mothers

The adolescent and young adults further described the talks with their mothers as often being one-sided- the parent did the talking and the daughter was expected to just follow instructions. In addition, the young women explained that their mothers would often use words or terms such as ‘you have grown old now’, ‘do not enter the kraal’, and ‘don’t sit on a chair or next to men’. All these terms according to the young women had both literal (i.e. they were expected to adhere to these instructions) and hidden meanings. For example, a kraal is an enclosure for cattle located within an African rural homestead. The hidden meaning to this dictum could be that adolescents were forbidden from having penetrative sex. Further, the young women explained that elders in previous generations used dictums as part of Intonjane (a custom that marks passage into adulthood) to give instructions of how one should behave as a young woman. The adolescents and young adults also communicated that they interpreted the dictums according to their own understanding, their mothers did not clearly explain the meaning behind these idioms.

She [her mother] said that now you have become older and that I should not enter the kraal, she said when you menstruate don’t enter the kraal, don’t drink milk and don’t take Amasi [curdled milk]. (Adolescents and young adults, Ntabankulu)

The discussions by participants on the use of dictums in place of sexual health education suggest a cultural shift. Where traditions such as Intonjane are often used to try and address young married women’s reproductive health problems (e.g. inability to conceive children) instead of helping them transition to adulthood. Therefore this may leave young women confused and unable to reconcile the challenges of their environment with the expectations that the parents have. At the same time, the lack of links between cultural values and modern life may leave young women vulnerable to risky behaviors and susceptible to abuse by men.

Yes like my aunts had it done now at an older age because they were sick and needed it. (Adolescents and young adults, Ntabankulu)

The participants explained that when it comes to addressing risky behaviors parents focused on avoiding pregnancy and did not address other relationship dynamics such as partner choice, decision-making and contraceptive choices. Further, the participants shared that the mothers were giving mixed messages because even though they prefaced their instruction with ‘do not have sex’ they also seemed to acknowledge that adolescents may become sexually active and therefore felt that they should warn against unintended consequences (i.e. pregnancy).

That when you sleep with a boy without using a condom or without taking contraceptives you will fall pregnant. (Adolescents and young adults, Ntabankulu)

### Adult women expect adolescents to be empowered by sexual education received in school

Themes emerged from the discussions of adult women (25–35 years of age) that indicated a link between being young parents to early adolescents and how they were socialized as teenagers. This input was important, as it gave an indication of how much had changed or remained stagnant over time in terms of rural sexual health communication. Adult women mainly explained that current adolescents have easy access to reproductive and sexual health information as compared to what they (adult women) received as adolescents.

They get these lessons form school, there is also this thing of ABC, these kids get educated in school, we also try to help them, but I see they do not care because I at least can see that they are growing up during the time where clinics are closer, everything comes easier. (Adult woman, Ngqeleni)

Since adult women had perceptions that adolescents receive adequate sex education in school, it was important to confirm this notion. In sharing the content of their sexual health education adolescents and young adults confirmed that they did receive sexual education in school as part of their Life Orientation subject. However, we noted that some schools gave basic information with varying content on the topics such as menstruation, HIV/AIDs and sexually transmitted infections (STIs), as well as condom use. Interestingly, the adolescents shared that messages from school also emphasized the importance of avoiding being in relationships with boys thus echoing messages given at home.

I have learned about condoms in grade 8 […] where we were told about it and that it is important to use it during sexual activities cause it can protect you from pregnancy and HIV, and STIs. (Adolescents and young adults, Ntabankulu)

We were told that STI are infections you get, like having a discharge, you get infected by sleeping with your boyfriend without using a condom. HIV/AIDS is also contracted by sleeping with your boyfriend without using a condom. You also get infected when you share needles. (Adolescents and young adults, Libode)

### How adult women received sexual health information in the past and how that influenced their behavior

We further drew from themes that emerged from the FGDs of adult women, where they reflected on their experiences of sexual health socialization. The adult women (in the 25–35 year FGDs) explained that when they were adolescents topics about sex were considered to be taboo. Many adult women intimated that they had to rely on peers for information, though often misguided, in order to have some understanding of what was happening to their bodies (e.g. menarche), the same applied to dealing with boy issues.

Honestly, for us, for me during those days when I first menstruated […] no one talked about those things [menstruation or sex], about contraceptives [pills, needles or condoms], no one even spoke about those things. (Adult woman, Ngqeleni)

Eeeh! Because when you are girls you chat and talk you see but that teaching you will not get at home. (Adult woman, Ngqeleni)

Further, primary health services were inaccessible at the time due to past political spatial organization of rural communities which led to women having to travel long distances to get any healthcare. The lack of access to these facilities really disempowered the teenagers of the time as they could have received some information and advice from the nurses as an alternative source. Even though the adult women reported that they felt they lacked sexual health knowledge and skills as teenagers, they also shared that being sexually active was the norm, even in the absence of any contraception options. What the then teenagers also considered important was to hide the relationships from their parents to avoid any punishment.

Eh, the thing is that in those days you see, for me to have my first child, what would happen, if you had a boyfriend […] what you would do, if there is a church [service] somewhere, or here in the rural community, you would pretend as if you have gone there and then leave with your boyfriend and go to his house and make sure you leave his house when it is still dark [early morning]. (Adult woman, Ngqeleni)

The discussions of adult women show that there has been a shift in terms of the information that the current adolescents receive, compared to what adult women did. However, their discussion also indicates that the fact that sexual health talks were impermissable in homes did not necessarily mean teenagers would abstain from being sexually active.

### How the experiences of being an adolescent inform sexual behavior

The current adolescents shared how their own socialization experiences shaped their sexual behavior. Adolescents and young adults intimated that even though at home they are given instructions to avoid boys and abstaining from sex, peers influenced their sexual behavior. Their discussions indicated that the majority of young women understood that they live in a time and culture where being sexually active is the norm, and is completely acceptable. Further, the young women shared that that it was up to an individual to decide when it was time to have sex and whatever was said at home did not matter much.

No one listens to her these days [mother’s instruction of abstinence] they just tell her in your times things were done a certain way, back then you used to wait [to have sex], what can I say. (Adolescents and young adults, Ntabankulu)

Most of the young and adult women who participated in the study further explained that they regarded the messages of abstinence as pointless. This indicated that the opinions of peers were valued since following what peers did was perceived as being good for ones reputation.

What are people [friends] going to say if I tell them that I do not have a boyfriend […] if they say wait at home, people look at you funny, you look like you are behind. (Adolescents and young adults, Ntabankulu)

Since the adolescents and young adults explained that they perceived it as a norm to be sexually active as a teenager, their attitudes towards safe sexual behavior also emerged. They expressed that they thought condom use was important, and also seemed to have an attitude that favored condom use. However, all the young women explained that whether one used condoms or not was up to the boyfriend. This response shows that many young women may not feel necessarily empowered or self-efficacious to use condoms. Using medical contraceptives would be an alternative to using condoms even though these forms do not protect against diseases (STIs and HIV/AIDS) at least pregnancy would be prevented. However, the young women seemed to have negative attitudes towards the use of these as well. When asked to explain their reasons, they shared that they have experienced and learned from peers that medical contraceptives have many side effects and therefore were not the best choice. It also seemed that in the rural communities it was important to have a body that peers approve of (‘your body should not shake’), this indicates that you take care of yourself as a young woman.

I was afraid, the other thing is, they [friends] told us that needle contraceptives are not the same, sometimes when you use a contraceptive it will not agree with you. They say you will have water … here [pointing at her private parts] or you will menstruate without stopping or your body will not be firm. (Adolescents and young adults, Ntabankulu)

## Discussion

This study aimed to gain an understanding of how adolescents, young and adult women from rural communities of the Eastern Cape received information concerning sexual health issues. The study also sought to understand how cultural practices and or school sexual health education programs influenced the socialization of young women on sexual issues. Further, the study drew from the experiences of young women to understand how socialization informed adolescents’ sexual behavior.

The findings show that it is mostly mothers who take the role of parenting, who also mainly carry out communication about sexual matters. Whereas the fathers are reported by participants to be mostly absent. This indicates that mothers have a big burden to carry, and this is a factor that may have serious implications on the well-being of the adolescents. Concerning the timing of the initial parent–child sexual health talk, adolescents and young adults shared that mothers waited for them to report menarche and then gave the talk. All the young women intimated that very few sexual health conversations took place after the initial talk. These findings are consistent with other studies in sub-Saharan Africa, which show that communication about sexual matters in low socio-economic communities tends to be poor especially with girl children (Bastien, Kajula, & Muhwezi, 2011; Delius & Glaser, 2002).

Adolescents and young adults also shared that they hold their mothers in high esteem, and that they expect them to give guidance through life issues. However, adolescents and young women also reported that it was not easy to talk to their mothers about emotional issues, and that it wouldn’t be easy to report things such as pregnancies as they would be scolded. This may be due to the perception that as parents, mothers have to be strict and harsh to influence good behavior (Juma, Alaii, Askew, Bartholomew, & Van den Borne, 2015). However, when parents and children are not closely connected and when warmth is not expressed in family relationships, opportunities are often missed. Open conversations can help parents cultivate trust between children and their parents and provide an opportunity to teach adolescents and young adults how to handle romantic relationships (Beckett et al., 2010; Wamoyi, Wight, & Remes, 2015).

The adolescents described the content of their talks with the parents as being one-sided and instructive rather than being educational. It also seemed like the parents focused on sexual abstinence and pregnancy avoidance. Very few adolescents and young adults shared that they were instructed to abstain from sex because of HIV infection risks or until they were in stable relationships. While discouraging pregnancy for physical and socio-economic well-being of the adolescents is important, HIV prevention is equally if not more important since it leads to more adverse outcomes. This is particularly important for young women as they are disproportionately affected by HIV/AIDS (Zembe, Townsend, Thorson, & Ekström, 2012).

Further, in their discussions adolescents and young adults also explained that when parents tried to teach about safe sexual behavior, they often used idioms. These idioms seemed to have traditional symbolisms which were not well explained and thus not very well understood. It may be that mothers were not having the conversations with adolescents because of embarrassment or because of their own socialization (Coetzee et al., 2014; Juma et al., 2015). However, the fact that parents still found it important to relay messages of sexual health through dictums is important. Ethnographic studies conducted in South African urban and rural communities show that sexual health communication was mainly carried out in organized peer groups (Delius & Glaser, 2002; Wood, Senn, Desmarais, Park, & Verberg, 2002). Peer groups were viewed as important structures where girls and boys would teach each other about sexuality whilst relying on the guidance of older relatives (Delius & Glaser, 2002; Reid & Walker, 2005). In addition, among amaXhosa and Basotho nations boys received formal teaching in initiation schools and girls received teachings through Intonjane when they approached menarche (Khau, 2012). During Intonjane the girl would be placed in a secluded room for about eight days to a month. In this time she would be expected to follow certain restrictions, which included not consuming dairy and meat, not sitting on a chair, not being in the company of males. Intonjane was also restricted from physically entering a kraal. The older women in the family (aunts) would take time to teach about responsibilities of being a woman, fertility, and marriage. For example, a future mother was to avoid dairy products as they are linked to her fertility (Cloete, 2015). She would also be taught sexual responsibility, the importance of obedience, and understanding boundaries. Older women also linked not entering a kraal physically to preserving virginity. In the past livestock was a measure of a man’s wealth and being a virgin was also valorized as it added to the wealth of the girl’s father (Rice, 2014). Adolescents explained that Intonjane is no longer practiced for young women but instead it is done for older married women with fertility issues. The reason may be that the custom is now seen as being out-dated, but it may also be that socio-economic factors do not allow as it is a costly exercise. Although the custom was also grounded on perpetuating gender inequalities, which enforced patriarchy and promoted women being treated as commodities it was also to prepare women for the next stages of their life, it may be important to invite older women to participate as stakeholders who would share their knowledge-capital in interventions that seek to empower young women. Our FGD discussions indicate that early stages of parent–child relationships do not promote active decision-making (autonomy) and as a result adolescents are more likely to source wrong information elsewhere. They may then use this wrong information to enact risky sexual behaviors.

Another possible reason behind parents giving limited information to adolescents may be the fact that they have the perception that adolescents get educated on these issues in school, as older women in the focus group discussion asserted. The adolescents shared that indeed they receive sexual health education in schools, however, the content does not seem to be uniform across the board. Further, many young women intimated that teachers often echo what is said at home without addressing any issues the teenagers may be facing or about how safe sexual behavior could be enacted. These results are similar to findings elsewhere, where the Life Orientation subject has been shown as being inadequate for empowering adolescents (Khau, 2012; Smith & Harrison, 2013). Even though teachers may be focused on fulfilling a mandate given by the department of education, the effects will be limited due to the variation in how the content is delivered. This is because sexual health is seen as a small component of a bigger Life Orientation subject. Also, teachers are not specifically trained to deliver the subject (DePalma & Francis, 2014). Literature shows that the education policy also gives autonomy to teachers on how the sexual health topic is delivered, meaning that information is provided on what teachers deem appropriate. Moreover, teachers often feel they have to be sensitive to social dynamics (e.g. culture and religion) and may thus avoid giving too much detail for fear of upsetting social structures (DePalma & Francis, 2014; Department of Education, 2008; Khau, 2012; Landor, Simons, Simons, Brody, & Gibbons, 2011).

Notwithstanding the negative characteristics of cultural customs, the findings indicate that there may be cross-cultural confusion where parents are not able to bridge the gap between traditional value systems and what their adolescents learn in school. The cross-cultural confusion possibly indicates that young women remain disempowered or stuck in a cycle of disadvantage, which may further exposes them to risky behaviors.

On the note of behavior, it seems that adolescents and young adults perceive being sexually active as the norm. The discussions indicated that at some point many adolescents just decide to have sex because peers see it as the thing to do. However, these statements were not accompanied by how practicing safety measures would be important once that decision is made. Older women shared similar statements about their time as adolescents, the difference, however, is that for them no sexual health education was provided in school. Again, this points back to the fact that the sexual health education received at home and in school is done so without considering the prevailing norms, beliefs and behaviors. This was indicated by the statements made by adolescents that they know how serious HIV/AIDS infection risk is, and that condom use is important, but still think that it is up to the boyfriend to decide whether or not a condom will be used.

In conclusion, the manner in which rural adolescents and young women are socialized remains very limited. This is because parents and schools only focus on promoting the avoidance of pregnancy and HIV/AIDS to the neglect of other factors that influence risky sexual behavior. Where cultural practice was used in the past, only idioms are used to relay messages of caution risky sexual behavior. Suggesting that rural families do not see the importance of adapting cultural practices to congruent with the modern times. Further, there is a strong indication that a majority of adolescents will be sexually active without being equipped with skills to protect themselves or to deal with consequences.

Therefore, this suggests that it would be important to firstly design interventions that will increase the awareness of parents towards the sexual behavior of adolescents. It may be important to also equip parents to be better communicators on sexual matters. This may help teenagers transition into adulthood smoothly but it would also be important to allow teenagers to have agency in the process. It also seems that there is a need to have an in-depth analysis of the school sexual health curriculum as well as the delivery methods to ensure that they are efficient in equipping teenagers who are still in school. It may also be important to design interventions that are grounded in theory because these have been shown to be more effective in facilitating behavior change. For example, parents and teachers could be equipped to empower adolescents through using the self-determination theory (SDT) framework. The SDT theory is grounded on three psychological needs namely autonomy, competence and relatedness and these have to be met to instil lasting behavior change (Ryan & Deci, 2000).

Some limitations of this study include the fact that the FGD participants were recruited through IYA, the selection may have been biased towards those that approve of tribal leadership and therefore the views expressed in the discussions may not be representative of all adolescents, young adults and adult women living in rural communities. Further, due to the fact that this sexual health domain was part of a broader needs analysis study, which covered overall health of young women, sexual communication may not have been discussed exhaustively. However, to ensure that topics were covered extensively, debriefing sessions were held by the research team after each FGD session.
